# The economic costs of alcohol consumption in Thailand, 2006

**DOI:** 10.1186/1471-2458-10-323

**Published:** 2010-06-09

**Authors:** Montarat Thavorncharoensap, Yot Teerawattananon, Jomkwan Yothasamut, Chanida Lertpitakpong, Khannika Thitiboonsuwan, Prapag Neramitpitagkul, Usa Chaikledkaew

**Affiliations:** 1 Health Intervention and Technology Assessment Program (HITAP), Ministry of Public Health, Thailand; 2 Department of Pharmacy, Faculty of Pharmacy, Mahidol University, Thailand; 3 International Health Policy Program (IHPP), Ministry of Public Health, Thailand

## Abstract

**Background:**

There is evidence that the adverse consequences of alcohol impose a substantial economic burden on societies worldwide. Given the lack of generalizability of study results across different settings, many attempts have been made to estimate the economic costs of alcohol for various settings; however, these have mostly been confined to industrialized countries. To our knowledge, there are a very limited number of well-designed studies which estimate the economic costs of alcohol consumption in developing countries, including Thailand. Therefore, this study aims to estimate these economic costs, in Thailand, 2006.

**Methods:**

This is a prevalence-based, cost-of-illness study. The estimated costs in this study included both direct and indirect costs. Direct costs included health care costs, costs of law enforcement, and costs of property damage due to road-traffic accidents. Indirect costs included costs of productivity loss due to premature mortality, and costs of reduced productivity due to absenteeism and presenteeism (reduced on-the-job productivity).

**Results:**

The total economic cost of alcohol consumption in Thailand in 2006 was estimated at 156,105.4 million baht (9,627 million US$ PPP) or about 1.99% of the total Gross Domestic Product (GDP). Indirect costs outweigh direct costs, representing 96% of the total cost. The largest cost attributable to alcohol consumption is that of productivity loss due to premature mortality (104,128 million baht/6,422 million US$ PPP), followed by cost of productivity loss due to reduced productivity (45,464.6 million baht/2,804 million US$ PPP), health care cost (5,491.2 million baht/339 million US$ PPP), cost of property damage as a result of road traffic accidents (779.4 million baht/48 million US$ PPP), and cost of law enforcement (242.4 million baht/15 million US$ PPP), respectively. The results from the sensitivity analysis revealed that the cost ranges from 115,160.4 million baht to 214,053.0 million baht (7,102.1 - 13,201 million US$ PPP) depending on the methods and assumptions employed.

**Conclusions:**

Alcohol imposes a substantial economic burden on Thai society, and according to these findings, the Thai government needs to pay significantly more attention to implementing more effective alcohol policies/interventions in order to reduce the negative consequences associated with alcohol.

## Background

There is evidence that suggests that alcohol consumption imposes a marked economic burden on society in several aspects including health care costs, costs of productivity loss, costs of property damage, costs of criminal justice and law enforcement etc. [1-4]. Furthermore, it has been found that this impact does not only affect individual drinkers but also non-drinkers who live in the same society. As a result, a lot of effort has been made to control the production and consumption of alcohol even though alcohol is considered lawful in most countries worldwide.

Estimating the economic costs of alcohol is particularly useful for policy makers, public health planners, and researchers. Not only can the cost estimates be used in facilitating the formulation of alcohol-related policies or interventions aimed at reducing the harm associated with alcohol drinking through more comprehensive cost-effectiveness or cost-benefit analyses, but they can also be used to draw the public's awareness to the negative economic impact of alcohol.

There has, however, been a notable lack of generalizability of study results across settings. This lack is due to several reasons such as differences in the economic and health care infrastructures, disparities between labour markets, and law court systems etc. While many attempts have been made to estimate the economic costs of alcohol for various settings, the aforementioned differences greatly affect these estimations. It is also worth noting that estimations, when they have been made, are mostly confined to industrialized nations [5-9]. Although discrepancies in the estimation methods and cost components included in these studies were found to limit a direct comparison across studies, the economic burden of alcohol in the 12 developed settings was found to be substantial, ranging from 0.45 - 5.44% of Gross Domestic Product (GDP) while cost per capita ranged from 85.53 US$ PPP to 1,012.21 US$ PPP (in 2007 value) [10].

To our knowledge, there was only one study that estimated the economic costs of alcohol consumption in a developing country [11]. In addition, there is no well-designed study which estimates the economic cost of alcohol consumption in Thailand, where an increasing trend of alcohol consumption has been observed [12]. This is surprising as it has been found that about 31% of the Thai adult population are classified as drinkers. The percentage of the Thai population classed as heavy drinkers (>40 g/day in male and >20 g/day in female) has been put at 16.6% in males and 2.1% in females. This study was the first attempt to provide an estimate of the economic costs of alcohol in Thailand from a societal viewpoint.

## Methods

A prevalence-based approach was used to estimate the economic costs of alcohol associated with past and current use in the given year, 2006. To improve the quality and comparability, where possible, the current international guidelines, namely *the "International guidelines for estimating the costs of substance abuse" *published by the World Health Organization (WHO) in 2003 [13] were followed. The counterfactual scenario employed in this study was "What costs would have been avoided in Thailand in 2006 if there had been no past or present alcohol consumption in Thai society".

In economic terms, social consequences are the sum of private and external consequences that can represent a cost or a benefit. Private alcohol consequences are the consequences accruing to the individuals engaged in the drinking activity while external consequences are consequences of an action by drinker (s) that affect others. In this study, only external costs, the costs affecting others were considered as the private costs made by informed and rational decision making of individuals do not justify government action for many reasons [13]. It should also be noted that if the perceived cost of drinker is less than actual cost, the difference between the two can be viewed as social costs; however, this was not accounted for in this study.

This study focused on the costs associated with the negative consequences of alcohol consumption and therefore, any benefits related to a moderate consumption, such as CHD, are excluded from the analysis. This exclusion is consistent with a gross cost methodology. Our rational for only including gross costs is that estimates of the cost of alcohol should not include partial consideration of benefits [14]. In addition, gross cost calculations were conducted to allow for comparisons with most previous studies since the recent systematic review [10] indicated that most studies on economic costs of alcohol consumption conducted gross cost estimations rather than net cost estimations. However, the impact on the results of the use of net costs, where the benefits of alcohol consumption on health care cost are included, is assessed in the sensitivity analysis.

Both direct costs and indirect costs were measured in this study. In fact, intangible costs (i.e. pain, suffering and dereavement) should be included but due to methodological limitations it was omitted by most studies [10] including this one. Four major cost components, namely health care costs, criminal justice and law enforcement costs, cost of property damage due to traffic accident, and cost of productivity loss due to premature mortality and reduced productivity were eventually examined in this study.

### Calculation of Alcohol Attributable Fraction (AAF)

Alcohol Attributable Fractions (AAF) were used to quantify the proportion of drinking-related harm, e.g. illness, injury, death and crime attributable to alcohol. There are two approaches in calculating AAF. The first and most straightforward method is to directly attribute alcohol use on the basis of case series studies in which alcohol's involvement is systematically investigated either by blood alcohol concentration, self-reported drinking or to use administration records in which alcohol consumption is reported. In this study, this approach was used to quantify the number of injuries, traffic accidents, and offences and crimes attributable to alcohol consumption. The second method for estimating an AAF is the "indirect" method whereby estimates of the Relative Risk (RR_j_) of particular disease compared to abstention are combined with the prevalence data of the number of persons consuming at different levels (P_j_), using the following equation [15,16], where j refers to the drinking level.

The AAF derived from this method, together with the number of deaths and health care episodes, was used to estimate the number of deaths and health care episodes attributable to alcohol consumption in this study. Since only the gross cost was estimated, only those diseases with a calculated AAF > 0 (alcohol is the risk factor) were taken into account.

It would generally be difficult to apply the AAF across different societies unless it could be claimed that the two settings under comparison are identical in terms of, for example, ethnicity, social culture, and health and economic infrastructures; thus, where possible, local data was used to derive the AAF. While the RRs identified in this study were obtained from several meta-analyses [17-19], the information concerning the prevalence of drinking was derived from the National Health Examination Survey 2003-2004 conducted by the Health Systems Research Institute Office [20]. In this study, the prevalence of alcohol drinking was classified into four different categories according to the average consumption of pure alcohol, measured in grams per day. These four groups are abstainers (no alcohol within last year), responsible drinking (women 0-19.9 g/day, men 0-39.9 g/day), hazardous drinking (women 20.0-39.9 g/day, men 40.0-59.9 g/day), and harmful drinking (women >40.0 g/day, men >60.0 g/day) [21]. Table 1 summarizes the AAFs used in this study.

**Table 1:** Alcohol Attributable Fractions (AAFs) used in this study for quantifying the proportion of harms attributable to alcohol.

Disease/condition	AAFs		ICD-10 Code	Source of information
			
	Male	Female		
Acute and chronic pancreatitis	0.36	0.10	K85, K861	Carroa G et al. 2000 [18]
HIV/AIDs	0.38	0.17	B20 - B24	Fisher JC et al 2007 [25]
Alcohol Abuse	1.00	1.00	F101	English DR et al 1995 [17]
Alcohol Cardiomyopathy	1.00	1.00	I426	English DR et al 1995 [17]
Alcohol Dependence	1.00	1.00	F102	English DR et al 1995 [17]
Alcohol Gastritis	1.00	1.00	K292	English DR et al 1995 [17]
Alcohol Polyneuropathy	1.00	1.00	G621	English DR et al 1995 [17]
Alcohol Psychosis	1.00	1.00	F100, F103, F109	English DR et al 1995 [17]
Cholethaiasis	-0.19	-0.07	K80	Gutjahr E et al. 2001 [19]
Chronic pancreatitis, Alcohol induced	1.00	1.00	K860	Carroa G et al. 2000 [18]
Cirrhosis of the liver	0.82	0.27	K70-K74	Rehm J et al 2004 [21]
Degenerative of nervous system	1.00	1.00	G312	Rehm J et al 2004 [21]
Diabetes mellitus	-0.06	-0.02	E10 - E14	Gutjahr E et al. 2001 [19]
Epilepsy	0.66	0.21	G40 - G41	Gutjahr E et al. 2001 [19]
Ethanol Toxicity	1.00	1.00	T510	English DR et al 1995 [17]
Excess alcohol blood level	1.00	1.00	R780	Rehm J et al 2004 [21]
Female breast cancer	0.00	0.05	C50	Rodolfo B et al 2001 [37]
Fetal Alcohol Damage	1.00	1.00	P043, Q860	English DR et al 1995 [17]
Hemorrhagic stoke	0.14	-0.07	I63 - I66	Reynolds K et al 2003 [38]
Hypertension	0.21	0.12	I10 - I15	Carroa G et al. 2000 [18]
Ischemic Heart Disease	-0.12	-0.06	I20 - I24, I251 - I259	Carroa G et al. 2000 [18]
Ischemic Stroke	-0.01	-0.11	I60 - I62	Reynolds K et al 2003 [38]
Laryngeal Cancer	0.68	0.26	C32	Gutjahr E et al. 2001 [19]
Liver Cancer	0.49	0.16	C22	Gutjahr E et al. 2001 [19]
Low Birth Weight	0.04	-0.02	P05-P07	Gutjahr E et al. 2001 [19]
Methanol Toxicity	1.00	1.00	T511	English DR et al 1995 [17]
Mouth and oropharynx Cancer	0.52	0.17	C00-C14	Gutjahr E et al. 2001 [19]
Oesophageal Cancer	0.60	0.24	C15	Gutjahr E et al. 2001 [19]
Oesophageal Varices	0.77	0.23	I85	Gutjahr E et al. 2001 [19]
Other Ethanol Poisoning	1.00	1.00	T512-518, T524, T598, X44, X46-X47, X64-X67, Y513, Y56, Y564, Y566, Y573	English DR et al 1995 [17]
Other neoplasms	0.14	0.04	D00-D48	Rehm J et al 2004 [21]
Psoriasis	0.38	0.17	L40	Gutjahr E et al. 2001 [19]
Stomach cancer	0.69	0.10	C16	English DR et al 1995 [17]
Supra Ventricular Cardiac Arrhythmia	0.39	0.16	I47-I49	Gutjahr E et al. 2001 [19]
Road injuries	0.36	0.36	V 01-V89, Y 85	Local information
Offences against Officials/Officers	0.23	0.23	-	Laixuthai A et al. 2001 [29]
Offences relating to fire	0.03	0.03	-	Laixuthai A et al. 2001 [29]
Offences of defamation	0.08	0.08	-	Laixuthai A et al. 2001 [29]
Offences relating to Sexuality	0.11-0.35	0.11-0.35	-	Laixuthai A et al. 2001 [29]
Offences against body and life	0.21	0.21	-	Laixuthai A et al. 2001 [29]
Offences against Property	0.02-0.05	0.02-0.05	-	Laixuthai A et al. 2001 [29]
Offences of Extortion, Blackmail, Robbery and Gang-Robbery	0.01-0.05	0.01-0.05	-	Laixuthai A et al. 2001 [29]
Offences relating to criminal damage	0.59	0.59	-	Laixuthai A et al. 2001 [29]
Offences relating to incursion	0.16	0.16	-	Laixuthai A et al. 2001 [29]
Violation of alcohol Act	1.000	1.000	-	Laixuthai A et al. 2001 [29]
Violation of road traffic Act	0.060	0.060	-	Laixuthai A et al. 2001 [29]
Road traffic accident	0.076	0.076	-	Police Information Technology Center 2006

### Direct costs

In this study, direct costs included health care costs, law enforcement costs, and costs of property damage due to traffic accidents.

### Health care cost

The first step in assessing health care costs was to estimate the number of patients in each disease category attributable to alcohol consumption by multiplying the AAF (see table 1) with the total number of patients suffering from the corresponding disease. This information was obtained from the Thai Burden of Disease (BOD) study [22]. In this study, the list of diseases included in the estimation was adapted from the WHO Global Burden of Disease Project [23] and identical to those used in the studies of Rehm et al. 2003 [24]and Jarl et al. 2008 [3]. There was, however, one exception, HIV/AIDS, which was included in this study. The reason for this inclusion is that there is empirical evidence demonstrating a significant relationship between alcohol drinking and unsafe-sex [25-27] and that HIV/AIDS is the important public health problem in Thailand with a high prevalence and mortality rate.

The costs associated with out-patient services were estimated by multiplying the number of patients suffering from a disease attributable to alcohol by the number of out-patient visits these patients paid per year. Specific out-patient unit costs were also factored in. In this study, the disease-specific annual figures for out-patient visits and unit costs were both derived from a database at the Center of Health Equity Monitoring, Faculty of Medicine, Naresuan University. This database includes more than 16 million out-patient records collected from 81 public hospitals in 18 provinces throughout the country.

Similar to out-patient costs, the costs associated with in-patient services were estimated as the sum of in-patient costs for each disease attributable to alcohol. The numbers of hospital admissions and in-patient unit costs were derived from the Central Office for Healthcare Information (COHI), which covers approximately 90% of all hospital admissions in the country. These calculations were also adjusted to allow for missing coverage rates. More details on the calculation of health care costs attributable to alcohol can be found in Neramitpitakul et al. 2009 [28].

### Law enforcement costs

#### Police cost

The police cost attributable to alcohol consumption was calculated as the product of the numbers of recorded crimes and offences attributable to alcohol in year 2006 and the unit cost per case. The numbers of crimes and offences brought to the attention of the police in 2006 was obtained from the Royal Thai Police Annual Report (2006). The unit cost was calculated using a top-down, or macro-costing technique, at two police stations in Thailand. The work time used in relation to crime and offences of the police is estimated at 25% of total work time. The unit cost per cases for crime and offences is, therefore, equal to 0.25 multiplied by the total cost (which is the summation of labour, material, and capital cost) in 2006 divided by the total number of crime and offences brought to the attention of police during the same year.

Information from a previous local study [29], in which police records for each arrest were retrospectively reviewed, was used to identify the proportion of crime and offences attributable to alcohol. In each arrest, police officers often assess and record whether alcohol was a factor in the arrest, based on the degree of visible intoxication of the suspect, the temporal and contextual data. The types of crimes and offences included in this study were crimes against property and violent crimes with AAFs of 0.03 and 0.31, respectively.

#### Court costs

Similar to police costs, court costs were estimated as the product of the number of offences as prosecuted by the criminal courts that were attributable to alcohol in the year 2006, and the unit cost. Information from the previous local study [29], in which court records for each case were retrospectively reviewed, was used to identify the proportion of crime and offences attributable to alcohol.

As shown in table 1, the offences included in the analysis were offences against officials, offences related to fire, violent crimes including assault, property crime, violation of the Alcohol Act, and violation of the Road Traffic Act. The total number of offences prosecuted by the courts was obtained from the Annual Report of the Office of Judiciary of Thailand 2006. Similar to the police costs, a macro-costing technique, using data from the Office of the Chief Judge of Region 1 and Office of the Chief Attorney of Region 1, was employed to estimate the unit cost.

### Cost of property damage due to traffic accidents

The cost of property damage due to road traffic accidents was estimated by multiplying the total monetary value of property damage due to road traffic accidents, obtained from the Department of Insurance, Ministry of Commerce, by the proportion of road traffic accidents attributable to alcohol (7.645%), derived from the Police Information Technology Center.

### Indirect costs

In this study, indirect costs included the costs of productivity loss due to premature mortality and reduced productivity due to both absenteeism (being absent from work) and presenteeism (reduced on-the-job productivity).

### Cost of productivity loss due to premature mortality

In this study, the Human Capital Approach [30] was used to estimate the costs of productivity loss due to premature mortality. In this approach, wages are assumed to be equivalent to the value of an individual's productive worth and used as a monetary conversion. The costs of productivity loss due to premature mortality was calculated as the products of the total number of deaths attributable to alcohol, by age and gender, and the present value of age and gender adjusted future earnings. A discount rate of 3% was applied. The number of deaths attributable to alcohol was calculated by combining the total number of deaths by age and gender with the gender and cause-specific AAFs (see table 1).

The average wage, classified by age and gender, was derived from the Socio-Economic Survey (SES) 2006 conducted by the National Statistic Office (NSO) while the age and gender adjusted life expectancies were obtained from the BOD project. Unlike in developed countries, Thailand's economy is still mainly dependant on agriculture, in which no formal age of retirement exists. Furthermore, it should be noted that in this study the number of lost working years for those who have died prematurely as the result of alcohol consumption was calculated by subtracting the mean age of death from the gender-adjusted life expectancies. However, age and gender- specific workforce participation rate and age and gender-specific income were taken into account in the calculation of the present value of future earnings so the individuals do not remain at the same level of productivity right up to the time of death.

### Cost of reduced productivity due to absenteeism and presenteeism (reduced on-the-job productivity)

To estimate the cost of productivity loss from absenteeism and presenteeism (reduced on-the-job productivity), a national cross-sectional survey was carried out in July 2006 using the sub-samples of the Socio-Economic Survey conducted by the National Statistics Office. A sample of the Thai population was interviewed. This sample came from 4,330 households, and included people aged between 15 - 60 years who were in paid employment. The questionnaire used in the survey included 1) general socio-economic information 2) work impairment due to health problems, which was adapted from the Work Productivity and Activity Impairment - General Health (WPAI-GH) questionnaire [31], and 3) recent patterns and profiles of alcohol consumption.

WPAI-GH composed of 6-items that asked about the impairment during the last seven days, as follows; 1) current employment status, 2) the number of hours missed due to health problems, 3) the number of hours missed due to other reasons, 4) the number of hours actually worked, 5) the degree health problem affected productivity while working, and 6) the degree health problem affected regular activities. The last two questions were evaluated on a scale of 11 points, ranging from 0 (no effect on work) to 10 (health problem prevent person from working). Questions regarding quantity and frequency of drinking during the past 30 days were asked. Pictures of various kinds of alcohol beverages and typical drinking sizes were also provided to the respondents. By using these questions, the mean percentages of absenteeism, presenteeism, and overall work impairment due to health problems could be calculated in each drinking category.

Based on the survey, the percentages of overall impairment (from both absenteeism and presenteeism) among former drinkers, responsible drinkers, and harmful drinkers was 5.6%, 1.7%, and 5.7%, respectively, were significantly higher than that of the abstainer. The multivariate analysis adjusted for the potential confounders was also conducted and shown in sensitivity analysis.

For each drinking category, the cost of productivity loss was then calculated by multiplying the excess impairment rate by the workforce participation rate, income per year and total number of drinkers in that category. In sensitivity analysis, a multivariate analysis using the Generalized Linear Model (GLM) was carried out to quantify the effect of alcohol drinking on productivity loss due to absenteeism and presenteeism (reduced-on-the job productivity) with adjustments made for age, gender, education and occupational factors. Also, the assumption of a 25% impairment rate among harmful drinkers was also employed in the sensitivity analysis.

All costs were presented in Thai baht, 2006. For inter-country comparison, costs can be converted into US$ using the Purchasing Power Parity exchange rate of 1US$ = 16.215 Thai baht [32]. The future costs were discounted at the rate of 3%, as recommended by the current health technology assessment guidelines in Thailand [33].

Sensitivity analyses were conducted to examine the extent to which the results are affected by the choice of methods or parameters used in the estimations. In more detail, the use of alternative discount rates (0% and 6%), alternative methods of calculating productivity loss due to absenteeism and presenteeism (multivariate analysis and assuming that 25% of productivity loss occurred among harmful drinkers), the opportunity loss from premature mortality of the non-working population (which was not included in the base-case analysis) was included in the analysis (this is performed by not taking into account the workforce participation rate when estimating the present value of age and gender adjusted future earnings), the different proportions of cases where alcohol was attributable to road traffic accidents (increases of 20% and 40% from the base case), the inclusion of health benefits from alcohol consumption, the exclusion of HIV/AIDS in the analysis, and the effect of introducing a retirement age of 60 in the estimations of premature mortality were extensively examined.

More details on methodology can be found in the technical report by Thavorncharoensap et al. 2008 [34].

## Results

The economic cost of alcohol consumption in Thailand in 2006 was estimated at approximately 156 billion baht, as shown in table 2. This represents 2, 392 baht per capita (approximately 143 $US PPP) or 1.99% of the national GDP. Indirect costs outweighed the direct costs, representing approximately 96% of the total cost. The largest cost component was the cost associated with productivity loss due to premature mortality and morbidity (95.8% of the total cost) followed by the health care costs (3.5%), cost of property damage due to road traffic accidents (0.5%), and costs associated with law enforcement (0.2%), respectively.

**Table 2:** Estimates of the economic cost of alcohol consumption in Thailand 2006

	Million baht * (2006)
***Direct cost***	
Health care cost	5,491.2
Law enforcement cost	242.4
- Court cost	156
- Police cost	86.4
Cost of property damage due to traffic accident	779.4
***Indirect cost ***	
Cost of productivity loss	
- Cost of productivity loss due to premature mortality	104,127.9
- Cost of productivity loss due to reduced productivity	45,464.6

**Total costs in million baht**	**156,105.4**

**Total cost as % of GDP**	**1.99**

**Total per capita (baht per capita)**	**2,391.3**

Total per capita ($US PPP per capita)*	**147.47**

Total costs in million $US (PPP) 2006*	**9627.22**

*16.215 baht = $US (PPP) 1

As shown in table 2, premature mortality accounted for 104,127.9 million baht. Meanwhile productivity lost due to absenteeism and presenteeism (reduced on-the-job productivity) stood at 45,464.6 million baht in Thailand in 2006. Health care costs were shown to be the largest direct cost component with an estimate of 5,491.2 million baht. The estimates for other direct cost components included the cost of property damage due to road traffic accidents (779.4 million baht), court costs (156 million baht) and police costs (86.4 million baht), respectively.

Concerning health care cost, the cost of out-patient service (2,488 million baht) was slightly lower than the in-patient costs (approximately 3,003 million baht, caused by about 220,000 hospital admissions). Males are associated with significantly higher costs for out-patient and in-patient services than females, as shown in table 3.

**Table 3:** Health care costs attributable to alcohol by types of service and gender

Type of service	Number *		Cost (Million baht)**		Total cost (Million baht)**
		
	Male	Female	Male	Female	
Out-patient department (OPD)	2.7	0.35	1,941.5	546.7	2,488.1
In-patient department (IPD)	0.17	0.05	2,324.9	678.2	3,003.1

Total	-		4,266.3	1,224.9	5,491.2

*Million person for OPD/Million visits for IPD

**16.215 baht = $US (PPP) 1

With respect to costs of law enforcement, there were approximately 22,000 and 16,000 crimes attributable to alcohol brought to attention of courts and police respectively, as shown in table 4. In addition, the unit cost for court and police were estimated at approximately 7,200 and 5,500 baht respectively.

**Table 4:** Cost of law enforcement attributable to alcohol

Source	Number of crime attributable to alcohol	Unit cost (baht)*	Total cost (Million baht)*
Court	21,709	7,190	156
Police	15,877	5,450	86.4

*16.215 baht = $US (PPP) 1

It was estimated that there were 39,460 premature deaths, or approximately 1.39 million potential life years lost, due to alcohol in 2006, as shown in table 5. For cost of reduced productivity as shown in table 6, males aged between 30 to 44 years old and 45 to 59 years old accounted for the highest cost of reduced productivity, respectively.

**Table 5:** Number of deaths and cost of premature death attributable to alcohol by gender

Number of death		Number of potential years lost		Cost of premature mortality (Million baht)*		Total cost (Million baht)*
	
Male	Female	Male	Female	Male	Female	
33,493	5,967	1,164,552	226,348	95,804.1	8,323.8	104,127.9

*16.215 baht = $US (PPP) 1

**Table 6:** Cost of productivity loss due to reduced productivity by gender and age

Age (Year)	Cost of productivity loss due to reduced productivity (Million Baht)	
	
	Male	Female
15 - 29	6,624.7	2,435.9
30 -44	18,885.8	4,819.2
45 - 59	10,288.6	2,410.5

Total	35,799.1	9,665.5

*16.215 baht = $US (PPP) 1

A number of sensitivity analyses were conducted to examine whether the results were sensitive to the changes of important assumptions or parameters. As shown in table 7, the total estimates ranged from 115,160.4 million baht to 214, 053.0 million baht. It was found that the inclusion of HIV/AIDS was the most significant possibly due to the high prevalence and high mortality rate of HIV/AIDS in Thailand. As this was the first study that included HIV/AIDS in the estimates, therefore, a sensitivity analysis would permit the comparison with other previous studies. The effect of introducing a retirement age of 60 years old in the calculation of premature mortality cost and choice of discount rate was also significant, as shown in table 7. Introducing a retirement age of 60 years old and choice of discount rate had a high impact on the results because they directly affected the costs due to productivity losses, which was the category with the largest alcohol-related cost.

**Table 7:** Results from sensitivity analyses

	Parameter/method	Total estimated (Million baht)
Discounting rate		
	0%	214,053.0
	3% (Base case)	156,105.4
	6%	126,311.4
Reduced productivity		
	25% impairment rate in harmful drinker	164,960.6
	Multivariate -Probabilistic Model	137,341.1
	Univariate (Base case)	156,105.4
Property loss due to traffic accident		
	AAF increases from base case 20%	156,261.3
	AAF increases from base case 40%	156,417.2
	Base case (AAF = 7.645%)	156,105.4
Premature mortality		
	Exclusion of non-labour worker (Base case)	156,105.4
	Inclusion of non-labour worker	182,695.9
Premature mortality		
	No effect of a retirement age(Base case)	156,105.4
	Effect of introducing a retirement age of 60	116,113.0
Health care cost		
	Gross cost (Base case)	156,105.4
	Net cost	155,271.6
Exclusion of HIV/AIDS		
	Inclusion of HIV/AIDS (Base case)	156,105.4
	Exclusion of HIV/AIDS	115,160.4

It was further shown that the methods of calculating productivity loss due to absenteeism and presenteeism (reduced on-the-job productivity) and the inclusion of the value of the non-working population fairly affected the total cost. When using the multivariate analysis to derive the excess impairment rate due to absenteeism and presenteeism (reduced on-the-job productivity), the total cost was estimated at 137, 341.4 million baht. However, when using an excess impairment rate of 25% among harmful drinkers, as is often quoted in literature [35], the estimated cost was slightly higher (164,960.6 million baht).

Concerning the inclusion of the value of the non-working population, e.g. housewives, in estimating the costs of premature mortality, it has been shown that when the value of the non-working population is taken into account, the total cost of alcohol increases from 156,105.4 million baht to 182, 659.9 million baht. The proportions of alcohol-related road traffic accidents, and the inclusion of health benefits from alcohol consumption was shown to have had a minimal effect on the total estimated cost.

## Discussion

Alcohol consumption has had a considerable negative impact on the economy of Thailand given that its social cost was equivalent to nearly 2% of the national GDP. These findings are similar to previous studies conducted in developed countries, although at the high end (see table 8). The indirect costs made up the largest cost component accounting for approximately 96% of the total cost, which is considered high, as compared to the previous studies [10]. This may be partly explained by the effect of workforce in the informal sectors and the inclusion of HIV/AIDS, as shown in the sensitivity analysis. In addition, the characteristics of health care systems as well as the underestimation and the exclusion of some direct costs (e.g. research and prevention, transfer cost, etc) also accounted for the high proportion of indirect cost in this study.

**Table 8:** Comparison of total cost of alcohol consumption estimates from various industrialized countries

Country/Year	% of GDP
Australia/2004-5 [39]	1.39
Canada/2002 [7]	1.2-1.5
France/1997 [2]	1.42
Sweden/2002 [3]	0.9-1.3
US/1985 [8]	1.66
Thailand/2006 (This study)	1.99

With respect to the health care system, unlike in more developed countries, Thailand is still facing problems of access to quality health care. As a result, life expectancy of patients is more likely to be shorter than their developed countries counterparts. The significant lower cost of treatment and higher mortality rate in Thailand would contribute to the higher proportion of indirect costs in this study.

Unlike other studies conducted in North American and Europe [5,8,9,13,23], this study found a relatively low proportion of costs associated with law enforcement and property damage. These findings may partly be explained by the fact that 1) cheaper labour costs in the judicial system and in repairing damaged properties were observed in Thailand, 2) the unit cost of judicial systems was underestimated, and 3) only criminal cases were included.

At present, there is no objective way of measuring alcohol-related crime as the crime committed by drinkers even when he or she is under the influence of alcohol does not necessarily mean that the crime can be ascribed to alcohol use. Therefore, the best way to measure alcohol- related crime is to use subjective information from suspects and the temporal and contextual data. However, this may be subjected to overestimation of the causal relationship.

As for some diseases, heavy alcohol consumption is associated with negative effects while low consumption is beneficial for health, the inclusion of only disease with AAF > 0 in the analysis will lead to an underestimation of the gross cost. However, this issue would minimally affect the current estimates, as the prevalence and AAF of diseases omitted from the analysis are very small.

Another limitation that could be pointed out is the fact that cost-of-illness and social costs studies can demonstrate the scale of problems, but they are limited in determining how resources are to be allocated because they do not measure the individual benefits or compare interventions in terms of their costs and outcomes. Nevertheless, an extension of this work in Thailand is the use of the estimates from this study to inform and to raise awareness of the public and to support the legislation of the recent Alcohol control Act.

Due to the unavailability of reliable data in the local setting some costs were omitted from the analysis. These were the costs of research and prevention, the costs administrating transfer costs, victim time cost, the costs of property damage due to fire and vandalism, and costs of incarceration. The economic costs of alcohol would have been higher if these omitted factors could have been incorporated into the analysis. However, as most of the major cost components were already included, the anticipated increase from an inclusion of these costs would not be large. It is noteworthy that this study focused only on tangible costs where the policy implications are considered, and that the inclusion of intangible costs would only reaffirm the significant impact of alcohol in the country.

Besides the exclusion of costs as indicated above, there were other limitations worthy of being addressed. Firstly, individual earnings, in terms of annual incomes used for estimating the cost of productivity loss due to morbidity and premature mortality in this study, were likely to be underestimated because they failed to include the fringe benefits, the value of housekeeping services, and work outside the labour market. Secondly, the cost of reduced productivity due to absenteeism and presenteeism (reduced on-the-job productivity) was calculated only for the workers aged between 15 and 60 years old, and thus excluded the productivity loss from the workers who were older and still working in the informal sector. Thirdly, the unit cost of law enforcement may also be underestimated since the cost of incarceration and the cost of probation were not included in the analysis. Lastly, it should be noted that the counterfactual scenario used in this study and other cost -of -illness studies was based on strong assumptions and so may not be entirely realistic.

The findings from this study can be utilized to inform public health administrators and researchers concerning the information gap and research needed which would be useful for planning future investment in data infrastructure. With recognition of the limitations of human and financial resources available for research in developing settings, it is crucial that research resources are efficiently invested in important areas that would significantly increase the robustness or precision of the study results. The resources should be deployed to cover the information gap that is used to estimate the major cost components rather than the smaller ones. For example, because the productivity loss due to the reduction of working efficiency attributable to alcohol was considered to be a significant part of the economic costs of alcohol in Thailand, instead of using the external information, frequently quoted as a 25% reduction in working efficiency derived from the US study in 1970 [35], the authors conducted a national survey to quantify the productivity loss due to absenteeism and presenteeism (reduced on-the-job productivity) among the Thai population. Estimating the cost of presenteeism (reduced on-the-job productivity) is a new contribution to this area of study; however, due to the short article format, its methods, results and discussion cannot be made in detail in this paper. The authors acknowledge the importance of the novelty of an estimation of presenteeism (reduced on-the-job productivity) and will report on it separately in one of the series papers.

Another strength of this study is that it included an estimation of health care and premature mortality costs attributable to alcohol related HIV infections. There are strong reasons to include HIV/AIDS as an alcohol related disease (see above references in method section) though it is rather new in alcohol studies. It was found that HIV/AIDS contributed significantly to health care costs (20% of total health care costs) and the costs of productivity loss due to premature mortality (38% of the total cost of premature mortality). Therefore, we recommend that HIV/AIDS should be included in other studies conducted in areas with moderate and high HIV prevalence.

According to the statistics, only 31% of the Thai population consume alcohol. This is a very low rate compared to other countries where costs of alcohol studies have been conducted. Given that the cost as a percentage of GDP in Thailand is about the same as other countries studied, this means that the cost per consumer in Thailand is incredibly high. This can be explained by several reasons including pattern of drinking, characteristic of health care and law enforcement system in Thailand, which are different from those of developed countries.

Concerning the drinking pattern, based on WHO global status report on alcohol 2004 [12], in Thailand, the percentages of drinkers among males is far higher than in females. Therefore, in countries where the proportion of male drinkers was significant higher and where males received significant higher wages, the indirect costs would be higher, as compared to countries where proportions of males and females are similar. Although the total consumption and total number of drinkers in Thailand is not large, as compared to those of western countries, where costs of alcohol have been conducted, the consumption per drinker is approximately the same while the pattern of drinking is more likely to be harmful in Thailand. This could possibly explain the high cost of alcohol in Thailand.

The law enforcement system, especially on drink driving policies, is ineffective in Thailand, as compared to those of the developed countries. As a result, there are a large number of injuries and deaths from drink driving, as compared to those of the developed countries. In fact, this study revealed that injuries and deaths from car accidents due to alcohol consumption were the first and second leading causes of health care costs and premature mortality costs, respectively.

With respect to health care system, unlike the developed countries, Thailand is still facing the problem of access to quality of health care. As a result, life expectancy of patients is more likely to be shorter than their developed countries counterparts. Even though the proportion of drinkers in Thailand was only 30%, the higher mortality rate would contribute to the high cost of total estimates per GDP.

According to the latest statistics from the Excise Department, Ministry of Finance, the excise tax on alcohol was 72,871 million baht in 2006 [36]. This study points out the fact that the economic cost associated with alcohol substantially exceeded its revenue generated for the Thai government. The excise tax accounted for only 47% of the economic cost of alcohol. However, it should be noted that the comparison of the tax revenue from alcohol and the societal costs may not be appropriate because this study did not use the Thai government's perspective. Also, there is the need to include many of the payments, such as compensation and bail, which are considered transfers from the societal viewpoint.

## Conclusions

Alcohol imposes a substantial economic burden on Thai society. Based on these findings, the Thai government needs to try to minimize the adverse effect of alcohol consumption for the benefit of society as a whole. In addition, it is hoped hat the findings provided will stimulate discussion and improvements on alcohol policies in Thailand.

## Competing interests

The authors declare that they have no competing interests.

## Authors' contributions

MT conceived of the study, participated in its design and coordination, and drafted the manuscript. YT conceived of the study, participated in its design and helped to draft the manuscript. JY involved in the analysis of the cost of law enforcement and help to draft the manuscript. CL and KT involved in the analysis of the indirect cost. PN involved in the analysis of health care cost. UC participated in its design and helped to draft the manuscript. All authors read and approved the final manuscript.

## Pre-publication history

The pre-publication history for this paper can be accessed here:

http://www.biomedcentral.com/1471-2458/10/323/prepub
